# Psychometric Evaluation of the Parental Reflective Functioning Questionnaire in Chinese Parents

**DOI:** 10.3389/fpsyg.2022.745184

**Published:** 2022-01-28

**Authors:** Panqin Ye, Jiawen Ju, Kejun Zheng, Junhua Dang, Yufang Bian

**Affiliations:** ^1^Collaborative Innovation Center of Assessment for Basic Education Quality, Beijing Normal University, Beijing, China; ^2^Department of Surgical Sciences, Faculty of Medicine, Uppsala University, Uppsala, Sweden; ^3^Child and Family Education Research Center, Beijing Normal University, Beijing, China; ^4^Institute of Mental Health and Education, Beijing Normal University, Beijing, China

**Keywords:** parental reflective functioning, scale adaptation, confirmatory factor analysis, measurement invariance, Chinese parents

## Abstract

Parental reflective functioning (PRF) is important for parenting and child development. To effectively assess PRF in Chinese parents, this study aimed to revise the Parental Reflective Functioning Questionnaire (PRFQ) for the Chinese context. The original Chinese version of the PRFQ (PRFQ-C) was revised by following psychometric validation procedures in a sample of Chinese parents (*N* = 2,021, 1,034 mothers and 987 fathers). A series of psychometric analyses, including confirmatory factor analysis (CFA), internal consistency reliability analysis, discriminant validity, and criterion-related validity analysis, and analysis for measurement invariance between mothers and fathers, were conducted. The CFA results indicated that the final 12-item, three-factor model had a good fit {*χ*^2^(49)  =  472.381; CFI = 0.929; TLI = 0.904; RMSEA = 0.065, 90%CI = [0.060, 0.071]}. The Chinese version of the PRFQ with 12 items (PRFQ-12C) showed satisfactory reliability (omega = 0.68–0.82), discriminant validity [heterotrait-monotrait (HTMT) values < 0.85], and criterion-related validity. The PRFQ-12C also had measurement invariance across mothers and fathers. In conclusion, the PRFQ-12C is psychometrically sound and can be applied in China.

## Introduction

Parental reflective functioning (PRF) has been defined as parents’ capacity to reflect upon their own and their children’s mental states underlying observed reactions in the context of the parent-child relationship (Slade, 2005). Parents with high PRF regard their children as psychological agents and understand children’s thoughts and feelings from children’s perspective (Sharp et al., 2008). In contrast, parents who lack PRF may not be able to take the perspective of their children and fully recognize their children’s mental states. Since Slade et al. (2005) formally proposed the concept of PRF, an increasing number of researchers have focused on relevant studies.

Parental reflective functioning plays an important role in parenting behaviors and child developmental outcomes. Specifically, PRF can foster positive parenting behaviors, including parental sensitivity, parental involvement, communication, and limit setting (Slade, 2005; Rostad and Whitaker, 2016). PRF can also inhibit negative parenting behaviors, including parental insensitivity, negativity, overcontroling, and intrusiveness (Fonagy et al., 2002; Stacks et al., 2014; Ensink et al., 2016; Borelli et al., 2017). More importantly, PRF has a significant effect on child development, such as child attachment security, social competence, and emotional and social adjustment (Fonagy et al., 1991a,b, 2002; Slade et al., 2005; Benbassat and Priel, 2012; Esbjørn et al., 2013; Ensink et al., 2017). For example, parents with high PRF were observed to have children with secure attachment, while those with low PRF were more likely to have children with insecure attachment (Grienenberger et al., 2005; Slade et al., 2005; Stacks et al., 2014). Meanwhile, researchers found that PRF was significantly associated with children’s emotional (e.g., anxiety; Esbjørn et al., 2013), cognitive (e.g., reflective functioning; Ensink et al., 2015; Rosso et al., 2015), social (e.g., self-perception and romantic relationships; Benbassat and Shulman, 2016), and behavioral functioning (e.g., aggression; Smaling et al., 2016, 2017). Therefore, it is necessary to assess PRF to better explore the potential value of PRF.

The measurement of PRF mainly includes interview methods, such as the Adult Attachment Interview (AAI; George et al., 1984, unpublished; Fonagy et al., 1998), the Parental Development Interview (PDI; Aber et al., 1985, unpublished; Slade et al., 2003, 2004, 2009, unpublished), and the Working Model of Child Interview (WMCI; Zeanah and Benoit, 1995), among which the PDI is the most widely used. The PDI directly assesses parents’ representations of their children, themselves as parents, and parent-child relationships by asking parents to describe some situations and feelings about parenting. Although semi-structured interviews can offer the best opportunity to capture PRF in depth, the interview method is relatively time-consuming, costly, and impractical to be used in large samples. Additionally, PRF is a multidimensional concept (Luyten et al., 2017), which is difficult to be captured by interview methods because of only a single global score. Therefore, a parent self-report questionnaire to assess PRF called the Parental Reflective Functioning Questionnaire (PRFQ) was developed by Luyten et al. (2017) in Belgium and the United Kingdom. The PRFQ is an 18-item questionnaire consisting of three dimensions. First, the pre-mentalization (PM) dimension addresses parents’ difficulty entering the subjective world of their children and their tendency to make maladaptive and malevolent attributions to their children. Second, the certainty about mental states (CMS) dimension assesses the extent to which parents believe that they understand their children’s mental states. Third, the interest in and curiosity about mental states (IC) dimension reflects parents’ genuine curiosity about the mental states that underlie their children’s behaviors. For example, these PRFQ subscales in the English version have been shown to be related with mothers’ tolerance of infant distress (Rutherford et al., 2013, 2015), emotion regulation (Schultheis et al., 2019), and executive functioning (Rutherford et al., 2018). Mothers’ CMS in the Italian version was associated with childhood obesity (Pazzagli et al., 2019). The pre-mentalization in the German version predicted sensitivity to distress in mothers with postpartum depression (Krink et al., 2018). Although the PRFQ was originally designed for parents of children 0–5 years of age (Luyten et al., 2017), the PRFQ has been validated in samples of parents with children over 5 years of age (Pazzagli et al., 2018; De Roo et al., 2019).

The PRFQ is an open-source questionnaire that is available in multiple versions online (e.g., English version and Italian version). At present, the PRFQ has been validated in some countries (i.e., Belgium, United Kingdom, Italy, Canada, and Korea), most of which support the three-dimensional structure while the specific items of each dimension are different. For example, the Italian version of the PRFQ has been proven to be a valid and reliable measure of PRF in mothers and fathers in Italy, and its Cronbach’s alpha coefficients increased appreciably after some items (item 6, item 11, and item 14) were excluded (Pazzagli et al., 2018). De Roo et al. (2019) found a clear three-factor structure without item 11 and item 18 in Canadian parents with children aged from 0 to 12 years. For the Korean sample, however, Lee et al. (2021) found that the pre-mentalization factor of the original PRFQ was not appropriate.

To the best of our knowledge, the Chinese version of the PRFQ has not been validated in China. However, PRF is also very important among Chinese parents. In traditional Chinese culture, filial piety of children and parental authority are emphasized (Ho, 1986; Luo et al., 2013). Within the Chinese parenting context, Chinese parents may exert strong parental control and harsh discipline to make children obey (Luo et al., 2013; Ng et al., 2014). In such situations, understanding children’s mental states and parents’ own mental states in raising children is critical for Chinese parents for PRF may avoid the potential negative interactions between parents and children. In addition, most families place great importance on children’s educational success in the current Chinese cultural context (Quach et al., 2015), which may lead to parental anxiety and parenting stress and then make it difficult for parents to enter the child’s inner world. Therefore, it is important to adapt the PRFQ for the Chinese context to facilitate related research on PRF.

The present study aimed to examine the psychometric properties of the PRFQ in a Chinese sample. First, we tested the three factors with the original 18 items of the PRFQ using confirmatory factor analysis (CFA). Second, we conducted reliability, discriminant validity, and criteria validity analyses. Studies have found that parents with high PRF showed positive parenting practices (Stacks et al., 2014; Rostad and Whitaker, 2016; Luyten et al., 2017) were more tolerant for distress of their children (Rutherford et al., 2013, 2015) and had less parenting stress (Luyten et al., 2017). Meanwhile, parents with high PRF can not only take a perspective from their children, but also understand each member of the family better, and thus improve the level of family functioning (Cooke et al., 2017). Therefore, the correlations between PRF, parental warmth, parenting stress, and family functioning were conducted in the criteria validity analysis. Finally, we examined the measurement invariance across mothers and fathers.

## Materials and Methods

### Participants

A total of 2,021 valid questionnaires (95.78% response rate) were collected (1,034 mothers and 987 fathers) from seven public primary schools in urban and rural areas of Beijing, China. No statistically significant differences were found in age (*t*_1860_ = 0.562, *p* > 0.05) and education (*t*_2066_ = −0.988, *p* > 0.05) between respondents and non-respondents. These parents were biological parents of their children, and all lived with their children. The mean age of the mothers was 30.57 ± 3.84 years old (range, 31–52 years). The mean age of the fathers was 30.41 ± 4.73 years old (range, 30–64 years). The mean age of their children was 10.26 ± 0.32 years old (range, 9–12 years). In the sample of mothers, 3.20% completed middle school or below, 38.04% completed high school, 45.40% completed university, and 13.36% had a master’s education or above. In the sample of fathers, 5.10% completed middle school or below, 38.47% completed high school, 33.60% completed university, and 22.83% had a master’s education or above. In total, 85.11% of the mothers and 98.78% of the fathers were employed.

### Procedures

This study was approved by the Institutional Review Board at the study institution. First, standardized instructions about the purpose of the study were delivered to the sampled schools. The sampled schools were willing to support the implementation of this study. Then, written informed consent was obtained from the students’ parents. Finally, the questionnaires were provided to the students in sealed envelopes, and the students took them home for their parents to fill out. The questionnaires were returned by the students in sealed envelopes after being completed. All participants completed the PRFQ, questionnaires measuring family functioning, parenting stress, and parental warmth, as well as some demographic questions.

### Instruments

#### Parental Reflective Functioning

The Parental Reflective Functioning Questionnaire (PRFQ) is an 18-item self-report assessment that quantifies the level of PRF based on three subscales: PM, CMS, and IC. Each subscale has six items. Each item is rated using a 7-point Likert-type scale, from 1 (strongly disagree) to 7 (strongly agree). The PM subscale is designed to capture nonmentalizing modes, which indicates that the respondent struggles to accurately understand and interpret the child’s mental experience (e.g., “When my child is being difficult, he or she does that just to annoy me”). The CMS subscale contains items that assess the degree to which parents are certain about their children’s mental states (e.g., “I can always predict what my child will do”). The IC subscale assesses parents’ interest in and curiosity about their children’s mental states (e.g., “I am often curious to find out how my child feels”). The Chinese version of the PRFQ (PRFQ-C) used in this study was downloaded from an open-source website.^1^

We checked the items one by one and slightly adjusted item 7 to make it easier for participants to read. The original phrasing of item 7 (i.e., “I find it hard to actively participate in make believe play with my child.”) was slightly modified (i.e., “I find it difficult to actively play role-playing or fantasy games with my child.”).

#### Family Functioning

Family functioning was examined with the General Functioning subscale (GFAD) of the McMaster Family Assessment Device (FAD), which is a popular self-report questionnaire on family functioning (Epstein et al., 1983). The GFAD consists of 12 items with four response categories: strongly agree, agree, disagree, and strongly disagree. The item scores are averaged into a general score of family functioning, with higher scores representing healthier family functioning. The Chinese version of the GFAD (Liu and He, 1999) was used in this study. The Cronbach’s alpha coefficient was 0.89 in the current study. The fitting indexes of the CFA were *χ*^2^(44) = 564.222; CFI = 0.951; TLI = 0.926; RMSEA = 0.076; 90%CI = [0.071, 0.082]; SRMR = 0.051.

#### Parenting Stress

Parenting stress was measured with the parenting distress subscale of the Parenting Stress Index Short Form (PSI-SF, Abidin, 1995) in this study. The parenting distress subscale consists of 12 items (e.g., “Feel that I cannot handle things.”). Responses are given on a 5-point Likert-type scale ranging from 1 (“strongly disagree”) to 5 (“strongly agree”). Higher scores represent higher parenting stress. The PSI-SF has been widely used in China with high internal consistency (Lin et al., 2015). The Cronbach’s alpha coefficient was 0.91 in this study. The fitting indexes of the CFA were *χ*^2^(48) = 617.271; CFI = 0.953; TLI = 0.935; RMSEA = 0.077, 90%CI = [0.071, 0.082]; SRMR = 0.038.

#### Parental Warmth

Parental warmth was assessed with an adaptation of the Child Rearing Practices Report (Block, 1981; Chen et al., 2000, 2005), which includes nine items (e.g., “My child and I have warm, intimate times together” and “I feel a child should be given comfort and understanding when he/she is scared or upset”). The measurement has been validated and shown to be appropriate for studies in Chinese culture (Chen et al., 2005). The parents were asked to rate each item on a 5-point Likert-type scale, from 1 (strongly disagree) to 5 (strongly agree). Higher scores indicate higher levels of self-rated parental warmth. The Cronbach’s alpha coefficient in this study was 0.82. The fitting indexes of the CFA were *χ*^2^(23) = 246.732; CFI = 0.974; TLI = 0.959; RMSEA = 0.069, 90%CI = [0.062, 0.077]; SRMR = 0.026.

### Data Analysis

Data analysis was conducted in SPSS Version 20.0, Mplus Version 8.3. The absolute value range of skewness is 0.08 to 1.70, and the absolute value range of kurtosis is 0.05 to 2.70. According to Curran et al. (1996), skewness absolute values of 0–2 and kurtosis absolute values of 0–7 can be considered sufficient normality. First, to test the factor structure of the original PRFQ-C in the sample of Chinese parents, CFA was conducted in Mplus 8.3 (Muthén and Muthén, 1998–2019) by means of maximum likelihood (ML) estimation, with missing data handled with the full information maximum likelihood. The factor loading of each item was examined in order to ensure that each item was reliable to the latent factor (Brown, 2015). Model fit was evaluated using root mean square error of approximation (RMSEA), comparative fit index (CFI), and Tucker–Lewis index (TLI), and an RMSEA below 0.08 and other indexes (e.g., CFI and TLI) above 0.9 indicated good model fit to the data (Bentler, 1990; Hu and Bentler, 1999). Besides, CFA was also conducted on other shortened versions of the PRFQ in the literature (Pazzagli et al., 2018; De Roo et al., 2019) to further verify the rationality of the modified PRFQ-C.

Second, to examine the reliability of the modified PRFQ-C, the omega (w) coefficients (Brunner et al., 2012) were reported for all subscales. Third, the discriminant validity of the modified PRFQ-C was tested through heterotrait-monotrait (HTMT) analysis (Henseler et al., 2015). In addition, correlation analysis on the relationship between the three factors of the modified PRFQ-C, the original PRFQ-C, family functioning, parenting stress, and parental warmth was conducted to examine the criterion validity of the PRFQ-C.

Finally, the measurement invariance of the PRFQ-C measurement model was tested across mothers and fathers using the multigroup CFA technique. In the current research, three models of measurement invariance were examined in hierarchical order: configural, metric, and scalar invariance (Vandenberg and Lance, 2000; Milfont and Fischer, 2010). The first model tested whether the same factor structure existed across parent gender groups (configural invariance). The second model investigated whether the factor loadings were equal across groups (metric invariance). The third model examined whether the item intercepts were equal across groups (scalar invariance) based on the metric invariance achieved. As the chi-square test is sensitive to large samples and even small differences in covariance matrices could result in a significant chi-square value, chi-square was not used as a model fit index, and Δ*χ*^2^ was not used as a criterion to detect measurement invariance (Cheung and Rensvold, 2002; Chen, 2007; Kline, 2015). Instead, measurement invariance was assessed by comparing ΔCFI, ΔTLI, and ΔRMSEA with the cutoff criteria (i.e., ΔCFI ≤ 0.01, ΔTLI ≤ 0.01, and ΔRMSEA ≤ 0.015) suggested by Chen (2007) and Cheung and Rensvold (2002).

## Results

### Confirmatory Factor Analysis

The factor structure of the original PRFQ-C was examined by CFA. The initial model had a poor fit to the data, *χ*^2^(132) = 2538.507; CFI = 0.762; TLI = 0.724; RMSEA = 0.095, 90%CI = [0.092, 0.098]; SRMR = 0.083. The items with low loadings (i.e., below 0.4, included item 11, item 10, and item 18) and the items with cross-loading (i.e., item 3, item 14, and item 12) were removed (Ford et al., 1986). The results of the factor analysis of the original PRFQ-C were shown in the Supplemental Material. One item at a time was deleted to determine if the model fit improved. The model fit significantly improved after these items were removed, *χ*^2^(51) = 604.489; CFI = 0.907; TLI = 0.879; RMSEA = 0.073, 90%CI = [0.068, 0.079]; SRMR = 0.049. The modification indexes (MIs) revealed the items with error covariance. For example, item 17 was related to item 15 and item 16. The model fit the data well after these two covariance’s were held in the model, *χ*^2^(49) = 472.381; CFI = 0.929; TLI = 0.904; RMSEA = 0.065, 90%CI = [0.060, 0.071]; SRMR = 0.044. Finally, with these modifications, the three-factor PRFQ-C with 12 items (PRFQ-12C) was formed. As shown in Table 1, all items had factor loadings higher than 0.40 in the final model.

**Table 1:** Results of the factor analysis of the PRFQ-12C and the reliability values.

Factors	Item	Standardized factor loadings	Omega *(w)*
Pre-mentalization (PM)	Item 1	0.51	0.68
	Item 4	0.48	
	Item 7	0.48	
	Item 13	0.47	
	Item 16	0.60	
Certainty about Mental States (CMS)	Item 2	0.71	0.82
	Item 5	0.80	
	Item 8	0.66	
	Item 17	0.62	
Interest and Curiosity in Mental States (IC)	Item 6	0.75	0.76
	Item 9	0.76	
	Item 15	0.64	

Besides, we also conducted a CFA of the PRFQ-C for a second-order three-factor model, a bi-factor model, and other shortened versions in the literature, i.e., the three-factor model with 16 items in De Roo et al. (2019) and the three-factor model with 15 items in Pazzagli et al. (2018). The CFA results were shown in Table 2, which indicated that the fist-order three-factor model of the PRFQ-12C with 12 items fit best.

**Table 2:** Confirmatory factor analysis of the PRFQ.

Models	*χ* ^2^	*df*	CFI	TLI	RMSEA [90% CI]	SRMR
M1	3000.385	132	0.717	0.672	0.104 [0.100, 0.107]	0.132
M2	1815.231	117	0.832	0.781	0.085 [0.081, 0.088]	0.070
M3	1675.681	101	0.826	0.793	0.088 [0.084, 0.092]	0.065
M4	1586.393	87	0.827	0.791	0.092 [0.088, 0.096]	0.066
M5	472.381	49	0.929	0.904	0.065 [0.060, 0.071]	0.044
M6	780.123	51	0.877	0.841	0.084 [0.079, 0.089]	0.092

*M1 was a second-order three-factor model with 18 items for the PRFQ-C; M2 was a bi-factor model with 18 items for the PRFQ-C; M3 was the first-order three-factor of the PRFQ with 16 items appeared in De Roo et al., 2019; M4 was the first-order three-factor of the PRFQ with 15 items appeared in Pazzagli et al., 2018; M5 was a first-order three-factor model with 12 items for the PRFQ-12C; and M6 was a second-order three-factor model with 12 items for the PRFQ-12C*.

### Reliability

The omega (w) was 0.68 for the PM subscale, 0.82 for the CMS subscale, and 0.76 for the IC subscale, respectively (see Table 1). The reliability coefficients were satisfactory for the subscales of the PRFQ-12C except for the PM subscale.

### Discriminant Validity and Criterion-Related Validity

Table 3 demonstrates that all the HTMT values were <0.85, establishing the discriminant validity for the three-factor model. Table 4 shows the correlation matrix between the three subscales of the PRFQ-12C and the original three subscales of the PRFQ-C. The correlation coefficients of the modified version and the original version were *r* = 0.95 for the PM subscale, *r* = 0.95 for the CMS subscale, and *r* = 0.88 for the IC subscale, respectively. Table 5 shows the correlation matrix between the PRFQ-12C and family functioning, parenting stress, and parental warmth. All the correlation coefficients were significant (*p*s < 0.01). Specifically, PM had moderate negative correlations with family functioning (*r* = −0.36) and parental warmth (*r* = −0.35) and positive correlations with parenting stress (*r* = 0.48). CMS and IC were positively correlated with family functioning (*r* = 0.27 and *r* = 0.29, respectively) and parental warmth (*r* = 0.42 and *r* = 0.42, respectively) and negatively correlated with parenting stress (*r* = −0.11 and *r* = −0.12, respectively).

**Table 3:** Test of discriminant validity for the three-factor of the PRFQ-12C.

	PM	CMS	IC
PM	–		
CMS	0.28	–	
IC	0.18	0.59	–

*PM, pre-mentalization; CMS, certainty about mental States; and IC, interest and curiosity in mental states*.

**Table 4:** Intercorrelations between the PRFQ-12C and the PRFQ-C.

	PRFQ-C: PM	PRFQ-C: CMS	PRFQ-C: IC
PRFQ-12C: PM	0.95^**^	−0.27^**^	−0.23^**^
PRFQ-12C: CMS	−0.14^**^	0.95^**^	0.54^**^
PRFQ-12C: IC	−0.04	0.47^**^	0.88^**^

*PM, pre-mentalization; CMS, certainty about mental states; and IC, interest and curiosity in mental states*.

** *p < 0.01.*

**Table 5:** Intercorrelations of the PRFQ-12C and FF, PS, and PW.

S. no.		1	2	3	4	5	6
1.	PM	–					
2.	CMS	−0.18^**^	–				
3.	IC	−0.10^**^	0.46^**^	–			
4.	FF	−0.36^**^	0.27^**^	0.29^**^	–		
5.	PS	0.48^**^	−0.11^**^	−0.12^**^	−0.45^**^	–	
6.	PW	−0.35^**^	0.42^**^	0.42^**^	0.39^**^	−0.30^**^	–

*PM, pre-mentalization; CMS, certainty about mental states; IC, interest and curiosity in mental states; FF, family functioning; PS, parenting stress; and PW, parental warmth*.

** *p < 0.01.*

### Measurement Invariance

First, goodness-of-fit statistics for mothers and fathers sample were *χ*^2^(49) = 264.754; CFI = 0.923; TLI = 0.896; RMSEA = 0.065, 90%CI = [0.058, 0.073]; SRMR = 0.046, and *χ*^2^(49) = 271.189; CFI = 0.926; TLI = 0.901; RMSEA = 0.068, 90%CI = [0.060, 0.076]; SRMR = 0.048, respectively. Then, invariance was tested across mothers and fathers (see Table 6). Testing for configural invariance showed adequate fit (*χ*^2^ = 535.943; df = 98; CFI = 0.924; TLI = 0.898; RMSEA = 0.067; 90%CI = [0.061, 0.072]; SRMR = 0.047). We then examined the factor loading invariance (*χ*^2^ = 544.117; df = 107; CFI = 0.925; TLI = 0.907; RMSEA = 0.064; 90%CI = [0.058, 0.069]; SRMR = 0.048). The factor loadings were invariant across mothers and fathers (ΔCFI = 0.001; ΔTLI = 0.009; ΔRMESA = 0.003). Finally, we examined the intercept invariance (*χ*^2^ = 565.681; df = 116; CFI = 0.922; TLI = 0.912; RMSEA = 0.062; 90%CI = [0.057, 0.067]; SRMR = 0.048). The intercept was invariant across mothers and fathers (ΔCFI = −0.003; ΔTLI = 0.005; ΔRMESA = 0.002).

**Table 6:** Summary of the goodness-of-fit indexes of the tests of gender invariance.

S. no.	Invariance models	*χ* ^2^	*df*	CFI/TLI	RMSEA [90% CI]/SRMR	Model comparison	ΔCFI	ΔTLI	ΔRMSEA
1.	Configural	535.943	98	0.924/0.898	0.067 [0.061, 0.072]/0.047	–	–	–	–
2.	Metric	544.117	107	0.925/0.907	0.064 [0.058, 0.069]/0.048	2 vs. 1	0.001	0.009	0.003
3.	Scalar	565.681	116	0.922/0.912	0.062 [0.057, 0.067]/0.048	3 vs. 2	−0.003	0.005	0.002

*∆*CFI, change in CFI value; *∆*TLI, change in TLI value; and *∆*RMSEA, change in RMSEA value.

## Discussion

This study aimed to adapt the PRFQ-C (Luyten et al., 2017) to the Chinese context and examine whether the PRFQ-C is a valid measure of PRF among Chinese parents. To provide empirical support for the PRFQ-C, factor structure was examined, reliability and validity analyses were conducted, and measurement invariance across mothers and fathers was tested.

### Factor Structure

In this study, the three-factor structure of the original PRFQ-C was evaluated through CFA. The results of the CFA showed that the original model fit was poor. Specifically, there were items with low loadings in each subscale, such as item 11, item 18, and item 10. The results regarding the low loadings of item 11 and item 18 were consistent with previous research (Pazzagli et al., 2018; De Roo et al., 2019). This may be because item 11 (i.e., “I can sometimes misunderstand the reactions of my child.”) and item 18 (i.e., “I believe there is no point in trying to guess what my child feels.”) are reverse-worded items, and their meanings are easily misunderstood (Weems and Onwuegbuzie, 2001). The meaning of item 10 (“My child sometimes gets sick to keep me from doing what I want to do”) in the Chinese version may just describe a realistic situation that Chinese parents encounter in real life when raising children. For example, when the child is sick, the parents need to stay with the child or take the child to see the doctor, so that the parents cannot go to work. The expression of item 10 in the Chinese version may not have reflected parents’ misunderstanding of their children’s mental states. Therefore, item 10 had a weak loading on the PM subscale. In addition, there were three items with high cross-loadings (i.e., item 3, item 14, and item 12) according to the modification indexes in the original model.

Meanwhile, discussion with psychological experts and interview with parents also showed that these items were redundant in expression. Therefore, they were removed. The model fit changed from poor to acceptable after those items with low loadings and cross-loadings were removed. Finally, the Chinese version of the PRFQ with 12 items (PRFQ-12C) was formed, in which the PM subscale contains five items, the CMS subscale has four items, and the IC subscale includes three items. The model fit improved when the error covariance’s between item 17 and items 16 and 15 were added to the final CFA model. Considering the meanings of those items, it is reasonable that item 17 (“I always know why my child acts the way he or she does”) was positively related to item 15 (“I try to understand the reasons why my child misbehaves”), and negatively related to item 16 (“Often, my child’s behavior is too confusing to bother figuring out”), because parents’ efforts to understand the reasons of their children’s behavior in general should lead to greater knowledge about why their children behave and thus less confusions. Besides, the dimension of CMS is negatively correlated with PM and positively correlated with IC (Cooke et al., 2017; Luyten et al., 2017), and items 17, 16, and 15 belong to the dimensions of CMS, PM, and IC, respectively. Therefore, it can be considered reasonable to add the corresponding error covariance.

In summary, the three-factor structure of the Chinese version of the PRFQ was verified in Chinese parents after removing some items, which was consistent with previous studies in Western countries (Luyten et al., 2017; Pazzagli et al., 2018; De Roo et al., 2019; Gordo et al., 2020) but different from the results in a Korean sample (Lee et al., 2021). The three-factor structure of the original PRFQ was not appropriate in the Korean sample even after removing some items as the modification indices suggested (Lee et al., 2021). There are some possible reasons for this result. First, although it has a collectivistic culture, in recent years, China has imported and integrated individualistic values from the Western world, which influences parents’ theories about raising children. Secondly, the sample in this study comprised parents with school-aged children, while Lee et al. (2021)’s research focused on Korean parents with children 0 to 5 years of age. The structure of PRF may be different in parents with children of different ages.

### Reliability

The omega (*w*) coefficients of the revised PRFQ-12C factors ranged from 0.68 to 0.82, which indicated good reliability except for the PM subscale (*w* = 0.68). Generally, values greater or equal to 0.60, 0.70, and 0.80 are considered as marginal, acceptable, and good, respectively, and a value less than 0.60 indicates insufficient (Barker et al., 1994). Therefore, the reliability of the PM subscale is marginally acceptable and cannot be improved by deleting items in the PM subscale. This is consistent with Pazzagli et al. (2018) that reported a Cronbach’s *α* coefficient less than 0.70 for the PM subscale (*α* = 0.67). Besides, the evaluation of the reliability of the scale cannot be completely dependent on this numerical standard and also depends on the operational definition of the variables themselves (Crutzen and Peters, 2015). We suspect that the construct measured by the PM subscale may have broad connotations and is not easy to be operationalized, which may result in a low reliability value. However, this is beyond the scope of the current paper. Future studies may need to pay attention to this issue.

### Discriminant Validity and Criterion-Related Validity

All the HTMT values of the PRFQ-12C were low (ranging from 0.18 to 0.59). HTMT values < 0.85 establish discriminant validity for the constructs (Henseler et al., 2015). The correlation coefficients between the three subscales of the PRFQ-12C and the original PRFQ-C were very high (ranging from 0.88 to 0.95, see Table 4), which indicated that they are conceptually equivalent. The significant correlation coefficients between all three subscales of the PRFQ-12C and family functioning, parenting stress, and parental warmth indicated that the PRFQ-12C has good empirical validity. Specifically, PM had negative correlations with family functioning, but CMS and IC had positive correlations with family functioning, which was consistent with previous studies (e.g., Cooke et al., 2017). The results suggest that both mothers and fathers who have difficulties understanding their children’s mental states might have more family problems and have poor family relationships; in contrast, parents who are interested in and curious about their children’s mental states and are certain about them have better family relationships.

PM was positively associated with parenting stress, but CMS and IC were negatively associated with parenting stress. Similarly, Rutherford et al. (2013, 2015) found that PM and IC were related to mothers’ stress tolerance in a study following the BSIM paradigm in which mothers were asked to soothe an inconsolable baby simulator. High PRF might be an important buffer for stress associated with parenting (Luyten et al., 2017). Parents who have high PRF could regulate their own mental states toward their children’s distress and thus have less parenting stress.

Finally, there was a significant correlation between PRF and parental warmth, with PM being negatively associated with parental warmth but CMS and IC being positively associated with parental warmth. In other words, parents with low PM, high CMS and IC showed warmth and love to their children. This result provided empirical evidence for the theory that PRF is the basis of parental sensitivity (Slade, 2005, 2007), such that parents who have a high level of PRF are able to identify children’s needs and respond to them appropriately and in a timely manner. Taken together, the significant correlations between the three factors of the PRFQ-12C and family functioning, parenting stress, and parental warmth described above supported the empirical validity of the PRFQ-12C.

### Measurement Invariance

To test the measurement invariance at three restrictive levels across fathers and mothers, configural, metric, and scalar models were compared. The findings indicated that there were no significant differences among the fit indexes of configural, metric, and scalar invariance across genders. Therefore, the Chinese parent sample data supported the full configural, full metric, and full scalar invariance models. These results revealed that the PRFQ-12C had the same factor structure, factor loadings, and item intercepts for the mother and the father groups. Thus, it was concluded that the PRFQ-12C can adequately measure PRF in both mothers and fathers. The three-factor PRFQ-12C can be used to compare the mean PRF scores of mothers and fathers.

## Implications and Limitations

The findings of the current study have important implications for both theoretical and practical settings. First, our results provide cross-cultural support for the three-factor structure of PRF in Chinese parents. Though within different cultural contexts of parenting, PRF acts as a universal capacity of parents in understanding children’s thoughts and feelings from children’s perspective. Moreover, the PRFQ-12C can serve as a valid and reliable measure in Chinese parents of school-aged children in nonclinical settings. Empirical studies and related programs on PRF are still in its infancy in China. Psychologists and practitioners may utilize the measure to in promoting PRF of Chinese fathers and mothers (i.e., intervention on PRF) and link it to children’s better development.

Although the current study provided some initial evidence for the cross-cultural applicability of the PRFQ, several limitations should be noted in the present study. First, the sample in this study comprised parents whose children were elementary school students. During this period, children face academic challenges which make it difficult for parents to truly understand their children’s mental states. Thus, it is of vital significance to study the PRF of parents with elementary school children (Hesketh et al., 2010; Borelli et al., 2016). However, parents of children of different ages may encounter different challenges and difficulties (Deater-Deckard, 2008). Future studies can be conducted with other samples which include parents of preschool and adolescent children for further verification. In addition, participants in this study were recruited from only one city in China. Future research may replicate our findings in other regions of China. Finally, though the sample size in this study was large, the present data were cross-sectional. Therefore, examining the test-retest reliability was not possible, which can be further verified in future studies.

## Conclusion

In conclusion, the present study revised the PRFQ for Chinese parents and validated its psychometric properties against various criteria. A three-factor structure with 12 items for the Chinese Version of the PRFQ (PRFQ-12C) was supported, which had relatively good internal consistency and was significantly correlated with family functioning, parenting stress, and parental warmth. The measurement model for the PRFQ-12C was found to be invariant for mothers and fathers. Therefore, we conclude that the PRFQ-12C is suitable for research with Chinese mothers and fathers.

## Data Availability Statement

The raw data supporting the conclusions of this article will be made available by the authors, without undue reservation.

## Ethics Statement

The studies involving human participants were reviewed and approved by the Ethics Review Committee of Beijing Normal University. The patients/participants provided their written informed consent to participate in this study.

## Author Contributions

PY and YB designed the study. PY performed the data analysis and interpretation and wrote the first draft of the manuscript. JJ, KZ, and JD contributed to the final manuscript. All authors approved the final manuscript.

## Funding

This work was supported by the National Office for Philosophy and Social Sciences (no. 20ZDA071) and the Swedish Research Council (2018-06664).

## Conflict of Interest

The authors declare that the research was conducted in the absence of any commercial or financial relationships that could be construed as a potential conflict of interest.

## Publisher’s Note

All claims expressed in this article are solely those of the authors and do not necessarily represent those of their affiliated organizations, or those of the publisher, the editors and the reviewers. Any product that may be evaluated in this article, or claim that may be made by its manufacturer, is not guaranteed or endorsed by the publisher.

## Footnotes

^1^ https://www.ucl.ac.uk/psychoanalysis/research/parental-reflective-functioning-questionnaire-prfq
