# Supplementary material for: Psychometric Evaluation of the Parental Reflective Functioning Questionnaire in Chinese Parents
Source: Front Psychol. 2022 Jan 28;13:745184. doi: 10.3389/fpsyg.2022.745184 (PMC8837268; doi:10.3389/fpsyg.2022.745184)
Supplement: Supplementary file 1 [file Table_1.docx]

**TABLE 1 |** Results of the Factor Analysis of the original PRFQ-C

| Factors | Item | Standardized  factor loadings |
| --- | --- | --- |
| Pre-mentalization (PM) | Item 1 | 0.51 |
|  | Item 4 | 0.49 |
|  | Item 7 | 0.48 |
|  | Item 10 | 0.23 |
|  | Item 13 | 0.48 |
|  | Item 16 | 0.60 |
| Certainty about Mental States (CMS) | Item 2 | 0.69 |
|  | Item 5 | 0.77 |
|  | Item 8 | 0.64 |
|  | Item 11 | -0.18 |
|  | Item 14 | 0.60 |
|  | Item 17 | 0.67 |
| Interest and Curiosity in Mental States (IC) | Item 3 | 0.72 |
|  | Item 6 | 0.65 |
|  | Item 9 | 0.65 |
|  | Item 12 | 0.48 |
|  | Item 15 | 0.71 |
|  | Item 18 | -0.34 |
